# Nanomaterials in targeting amyloid-β oligomers: current advances and future directions for Alzheimer's disease diagnosis and therapy

**DOI:** 10.3762/bjnano.16.44

**Published:** 2025-04-22

**Authors:** Shiwani Randhawa, Trilok Chand Saini, Manik Bathla, Rahul Bhardwaj, Rubina Dhiman, Amitabha Acharya

**Affiliations:** 1 Biotechnology Division, C.S.I.R – Institute of Himalayan Bioresource Technology, Palampur, Himachal Prasesh, 176061, India; 2 Academy of Scientific and Innovative Research (AcSIR), Ghaziabad, Uttar Pradesh, 201002, India

**Keywords:** Alzheimer’s disease, amyloid, amyloid-β oligomers, detection, dissociation, nanomaterials

## Abstract

The amyloid cascade hypothesis posits that amyloid-β oligomers (AβOs) are the most neurotoxic species in Alzheimer’s disease (AD). These oligomers, characterized by their high β-sheet content, have been shown to significantly disrupt cell membranes, induce local inflammation, and impair autophagy processes, which collectively contribute to neuronal loss. As such, targeting AβOs specifically, rather than solely focusing on amyloid-β fibrils (AβFs), may offer a more effective therapeutic approach for AD. Recent advances in detection and diagnosis have emphasized the importance of accurately identifying AβOs in patient samples, enhancing the potential for timely intervention. In recent years, nanomaterials (NMs) have emerged as promising agents for addressing AβOs regarding their multivalent interactions, which can more effectively detect and inhibit AβO formation. This review provides an in-depth analysis of various nanochaperones developed to target AβOs, detailing their mechanisms of action and therapeutic potential via focusing on two main strategies, namely, disruption of AβOs through direct interaction and the inhibition of AβO nucleation by binding to intermediates of the oligomerization process. Evidence from in vivo studies indicate that NMs hold promise for ameliorating AD symptoms. Additionally, the review explores the different interaction mechanisms through which nanoparticles exhibit their inhibitory effects on AβOs, providing insights into their potential for clinical application. This comprehensive overview highlights the current advancements in NM-based therapies for AD and outlines future research directions aimed at optimizing these innovative treatments.

## Review

### Introduction

**Figure 1:**
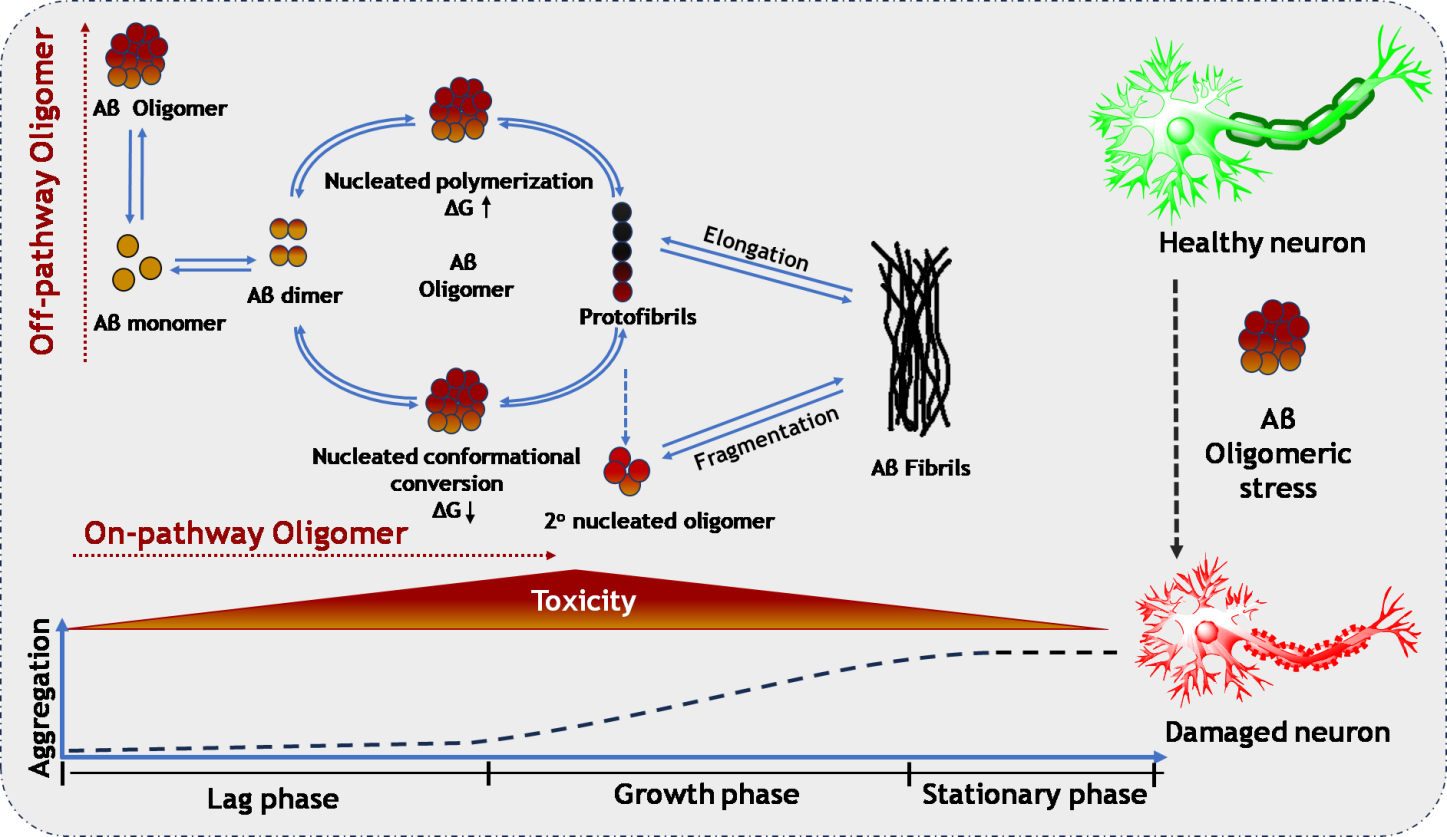
Schematic representation of the hypothesized pathways leading to the formation of toxic amyloid-β oligomers and their detrimental effects on neuronal health. The diagram outlines the key steps in the aggregation of amyloid-β peptides into oligomers, highlighting the underlying model mechanisms that contribute to neurotoxicity. (Figure 1 was redrawn from [11] using Microsoft PowerPoint and ChemDraw Professional (Version 20.1.1.125)).

The etiology of Alzheimer’s disease (AD) has traditionally been linked to the presence of amyloid-β 42 (Aβ42), a protein widely recognized as a key marker of the disease. However, a growing body of recent scientific evidence suggests that it may be the amyloid oligomers with smaller molecular weight, rather than the more conspicuous amyloid fibrils, that play a pivotal role in the development and progression of various protein misfolding diseases, including neurodegenerative disorders and type-II diabetes. Numerous studies have highlighted a disconnect between the accumulation of amyloid plaques observed in post-mortem examinations and the neurological deficits experienced by patients during their lives. To explain this lack of correlation, multiple hypotheses have been proposed. Among these, the “oligomer hypothesis” has recently emerged as a leading explanation, positing that the toxic effects of these small oligomers may be more critical to the pathology of AD than the larger aggregated plaques. This shift in focus highlights the need for a deeper understanding of how these oligomers contribute to the disease process [1–2]. The proposition regarding amyloid oligomers has garnered significant attention over time, primarily because of three key observations related to drug candidates for AD therapy. These observations are (i) ineffectiveness of plaque-targeting therapies, that is, therapeutic agents that focus solely on the removal of amyloid plaques or amyloid fibrils have not demonstrated substantial improvements in patients’ cognitive behaviors; (ii) efficacy of oligomer-targeting drugs, that is, drug candidates that specifically target amyloid-β oligomers (AβOs) have shown greater clinical effectiveness in treating AD patients; and (iii) influence of the APOE4 allele, that is, positive clinical trial outcomes tend to have a higher concentration of AβOs in the brain of individuals carrying the E4 allele of apolipoprotein E (APOE4). The origins of AβOs in AD patients remain a subject of debate and require further extensive research for a definitive understanding. Genetic studies on AD patients indicate that mutations in the amyloid precursor protein (APP), such as the Osaka [3] and Arctic mutations [4], lead to an overproduction of soluble AβOs. These mutations are associated with an earlier onset of AD, often occurring before the age of 50 [5], suggesting that certain genetic factors can significantly accelerate the development of the disease. In contrast, the Icelandic mutations appear to have a protective effect, reducing both the overall levels of AβOs and the concentrations of amyloid fibrils, which are another form of amyloid aggregation linked to AD [6]. Additionally, the presence of the APOE4 genotype is notable, as it is found in approximately 65% of AD patients [5]. This genotype is associated with an increased tendency for amyloid monomers to aggregate into AβOs, potentially contributing to the pathology of the disease [7]. Comparisons between AD patients who carry the APOE4 allele and those who do not reveal that the former group has about three times the concentration of AβOs in the brain. This suggests that the APOE4 genotype plays a significant role in the progression of AD by facilitating the accumulation of these toxic oligomers [8]. AβOs exhibit several distinct characteristics that set them apart from amyloid-β fibrils (AβFs). They are small, globular aggregates that display a metastable and transient nature, along with a higher content of β-sheet structures [9]. These small protein aggregates can arise from specific interactions between *n*-mers (oligomers formed from a defined number of monomers), or from non-specific interactions, akin to micelles. The precise mechanisms underlying the formation of AβOs during the growth of AβFs remain elusive. However, researchers have identified three main pathways to explain this process, as illustrated in Figure 1. The first two pathways fall under the “on-pathway” model, which includes nucleated polymerization and nucleated conformational conversion. These models suggest that oligomers are transient species that form as intermediates during the transition from monomers to mature AβFs. In this context, AβOs are considered stepping stones on the pathway to fibril formation. In contrast, the third model, known as the “off-pathway” formation of AβOs, asserts that oligomers represent a separate class of aggregates that do not progress to form fibrils. This model highlights the possibility that AβOs may have distinct properties and biological implications that differ from those of amyloid fibrils, suggesting a more complex relationship in the pathology of amyloid-related diseases. Understanding these pathways is crucial for unraveling the role of AβOs in the development and progression of AD [10].

Numerous studies have indicated a strong association between neuronal dysfunction and the presence of oligomeric species. Researchers are increasingly coming to a consensus that the neurotoxicity observed in neurodegenerative diseases (NDs) is not attributable to a single, isolated toxic conformer of amyloid oligomers. Instead, it appears to be the result of a diverse and heterogeneous population of oligomers. Understanding the factors that influence their toxic nature is crucial for developing tools for early detection and treatment. Despite years of research, targeting AβOs has yielded limited clinical success, primarily because of the tendency to treat patients at later stages, when extensive brain damage has already occurred. However, monoclonal antibodies targeting AβOs such as aducanumab, have demonstrated promising efficacy, leading to its FDA approval [12].

Early diagnosis of AD by targeting AβOs is crucial for improving outcomes. Current imaging methods, such as computed tomography (CT)/positron emission tomography (PET) with FDA-approved ^18^F-radiotracers (e.g., Amyvid™ and Tauvid™), detect plaques and tau tangles but not AβOs [13]. There is an urgent need for agents targeting AβOs to enable earlier and more accurate diagnosis and treatment. Despite extensive research, most AD treatments only offer temporary symptom relief and fail to target the root causes of the disease. This limited effectiveness stems from AD’s complex and multifactorial nature, which complicates early detection and the identification of reliable biomarkers and therapeutic targets. Moreover, the blood–brain barrier (BBB) poses a significant obstacle to effective drug delivery, further hindering the development of successful treatments.

Nanomaterials (NMs) offer promising solutions for the early detection and treatment of AβOs in AD. Because of their nanoscale size, NMs can interact with biological systems in ways that traditional treatments cannot. Their unique properties such as high surface area, quantum effects, and specific physicochemical traits make them ideal for developing advanced biosensors for early diagnosis and improving the sensitivity of AβO detection. In imaging, nanoparticles (NPs) can help to visualize localized protein accumulation, complementing existing diagnostic methods. Materials such as carbon-based NMs (e.g., graphene oxide) and metal NPs (e.g., gold and silver) enhance imaging sensitivity because of their distinct electrical or photoluminescent properties. For treatment, NPs can serve as drug carriers, improving delivery across the BBB and reducing side effects. Their large surface area allows for controlled drug release and targeted therapy, enhancing treatment efficacy. Additionally, NMs can interact directly with tissues and cells, potentially halting disease progression by preventing protein misfolding and the formation of toxic oligomers, a hallmark of AD pathology. Overall, nanotechnology holds significant potential to advance both the diagnosis and treatment of AβO-driven AD [14], and we will discuss these topics in the following.

### Mechanisms of neuronal cell toxicity induced by AβOs

Research has shown that AβOs possess a remarkable ability to penetrate cell membranes, largely due to their capacity to form porins within the lipid bilayer. This ability arises from the increased presence of β-sheet structures in AβOs, which can create distinct rafts in the membrane. These ring-shaped oligomers adhere to the cell membrane and inflict damage either by directly penetrating the membrane or by aggregating into fibrils that disrupt cellular integrity. Once internalized, AβOs activate *N*-methyl-ᴅ-aspartate-type glutamate receptors (NMDARs) located on neuronal membranes. This activation triggers endoplasmic reticulum (ER) stress through the stimulation of phospholipase C, leading to an influx of calcium ions (Ca^2+^) into the cytosol [15]. Elevated Ca^2+^ levels result in the accumulation of reactive oxygen species (ROS) and reactive nitrogen species, contributing to oxidative stress within the cell [16]. The increase in cytosolic Ca^2+^ also promotes the phosphorylation of ATP proteins, which, in turn, leads to the enhanced production of Aβ42 and AβOs, creating a vicious cycle. This cascade ultimately results in further spikes in intracellular Ca^2+^ concentrations sourced from the ER, which is linked to memory impairments commonly associated with AD. Additionally, elevated cytosolic Ca^2+^ activates the enzyme calcineurin, which is implicated in the activation of the Bcl-2-associated death promoter (BAD). This process, coupled with oxidative stress pathways originating from mitochondrial dysfunction, facilitates the release of cytochrome c from the mitochondria. This release is a key event that promotes caspase activation, initiating pro-apoptotic signaling that drives neuronal apoptosis [17]. Furthermore, AβOs can disrupt the membranes of endosomes and lysosomes, exacerbating neuronal cell death [18].

**Figure 2:**
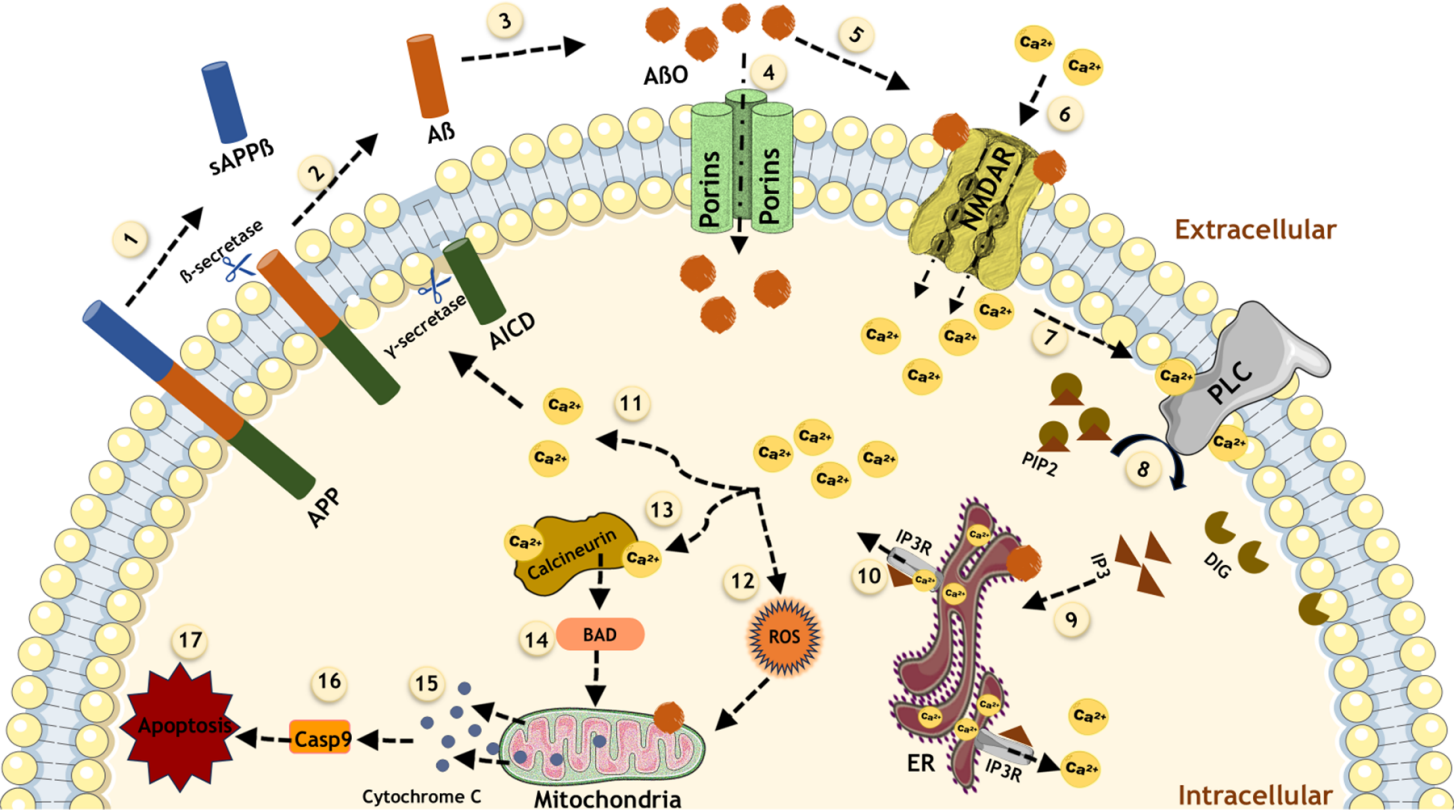
Illustration of calcium-mediated toxicity induced by oligomers. Oligomers initiate a calcium cascade that results in a series of harmful effects on cellular health. Elevated intracellular calcium levels activate phospholipase C (PLC), triggering endoplasmic reticulum (ER) stress and additional calcium release. This positive feedback loop exacerbates calcium dysregulation, leading to increased amyloid-β formation, oxidative stress, mitochondrial dysfunction, and ultimately apoptosis. (Figure 2 was created using Microsoft PowerPoint and Servier Medical Art (https://smart.servier.com)).

In another dimension, AβOs exhibit a strong affinity for cellular prion protein (PrPC) receptors, binding to them irreversibly. The formation of the oligomer–PrPC complex, together with the co-activation of the mGluR5 receptor, leads to the activation of intracellular Fyn kinase. This activation causes dysregulation of calcium ion homeostasis, hyperphosphorylation of tau protein, and disruption of synaptic functions. Together, these processes, as depicted in Figure 2, contribute significantly to the neurodegenerative pathways associated with AD, highlighting the multifaceted role of AβOs in neuronal dysfunction and cell death [19].

### Conventional methods for addressing the presence and toxicity of AβOs

AβOs are small aggregates formed from the misfolding and aggregation of amyloid-β (Aβ) peptides, primarily Aβ40 and Aβ42. These oligomers typically consist of a limited number of Aβ monomers, often ranging from trimers to tetramers, but they can form larger aggregates under certain conditions. Their small size and unique structural properties contribute to several challenges in therapeutic targeting. They are considerably smaller than fibrillar aggregates and plaques, making them difficult to target with conventional binding agents. AβOs exhibit significant heterogeneity in size and conformation. This variability means that a single therapeutic agent may not effectively recognize all oligomeric forms, complicating the development of broad-spectrum therapies. Unlike larger aggregates, which may present multiple binding sites, AβOs have fewer defined surface characteristics that can be targeted. AβOs can interconvert between different oligomeric states and may also exist in equilibrium with monomeric and fibrillar forms. This dynamic nature poses a challenge for therapies that rely on specific binding, as the target may change rapidly in response to environmental factors or therapeutic intervention. As a result, the predominant strategies for targeting AβOs have largely been confined to biologics, particularly monoclonal and polyclonal antibodies. The advantages of using antibodies stem from their remarkable capacity to recognize conformational epitopes that are unique to various oligomeric forms, thereby facilitating the selective targeting of pathogenic AβOs.

The most widely utilized monoclonal antibodies in AD research are 6E10 and 4G8 [19–21]. These antibodies were generated by immunizing mice with specific peptide fragments of Aβ, allowing them to bind effectively to amyloid aggregates. Importantly, the development of “conformation-dependent” antibodies, such as A11 and OC, marked a significant advancement in the field, as they were among the first to differentiate between AβOs and AβFs. This distinction is crucial for understanding the pathophysiology of AD, as oligomers are believed to be more toxic than fibrils [22–24].

In addition, the polyclonal antibody M94 has demonstrated high selectivity towards pathogenic AβOs, while not recognizing physiological Aβ monomers. This selectivity is vital, as it helps to minimize potential off-target effects and enhances the therapeutic profile of the antibodies [25]. Additionally, the monoclonal antibody mAb158 selectively targets soluble AβOs, including protofibrils, rather than monomeric Aβ or APP, highlighting its specific ability to focus on oligomers [26–28]. Recent developments have introduced novel approaches to target AβOs, such as the work by Haynes et al., who reported the creation of a unique anti-soluble AβO (E3) nanobody derived from an alpaca immunized with soluble AβOs. This E3 nanobody, conjugated with carboxyfluorescein (FAM), demonstrated effective recognition of both soluble AβOs and Aβ plaques, highlighting the potential for nanobody technology to complement traditional antibody approaches [29]. Conventional methods for targeting AβOs primarily rely on the use of antibodies because of their ability to recognize specific conformational epitopes associated with oligomers. The development of monoclonal and polyclonal antibodies has significantly advanced our understanding of AβOs in AD, allowing for targeted therapeutic and diagnostic strategies. While traditional antibody-based approaches remain valuable, ongoing innovations, such as the development of nanobodies and nanochaperones, promise to enhance the specificity and efficacy of oligomer targeting. Ongoing research into traditional therapeutic approaches, such as small molecules/compounds, immunotherapy, peptidomimetics, and chaperon proteins, as outlined in Table 1, continues to be a critical part of the effort to tackle AD. These conventional strategies, which target different aspects of AβO formation and accumulation, have shown promise in managing AD pathology. However, they face significant challenges, including limited efficacy and specificity in targeting the diverse forms of AβOs. By combining the strengths of both established and new technologies, researchers aim to develop more effective therapies for AD that can specifically target AβOs and ultimately slow or halt the progression of the disease.

**Table 1:** Classification of therapeutic strategies targeting AβOs and their mechanisms of action.

Class	Compound	Target	Mechanism of action	Reference

immunotherapy	aducanumab	AβOs and AβFs	aducanumab interacts with the area covering residues 3–7 in the N-terminal region of Aβ	[30]
	crenezumab	Aβ aggregates (including AβOs)	crenezumab, a fully humanized IgG4 monoclonal antibody, reduces the activation of Fc-gamma receptors (FcγRs) while preserving FcγR-mediated microglial phagocytosis and facilitating the clearance of AβOs	[31]
	gantenerumab	AβOs, plaque, and AβFs	mechanism of action involves binding with high affinity to both the N-terminal and central regions of Aβ peptides	[32]
	bapineuzumab	soluble AβOs and AβFs	changes in APOE4 carrier expression	[33]
	PMN310	AβOs	humanized PMN310 inhibits AβO-induced memory impairment and diminished synaptic loss and inflammation	[34]

small molecule/compounds	curcumin	AβOs, plaque, and AβFs	curcumin directly interacts with small amyloid species to inhibit aggregation and fibril production both in vitro and in vivo	[35]
	epigallocatechin gallate (EGCG)	AβOs and AβFs	the interactions of EGCG are determined by hydrophobic π–π and hydrophilic interactions with the aromatic side chains and Aβ backbone, respectively	[36]
	melatonin	AβOs	melatonin treatment inhibits the Aβ1–42-induced decline in Notch1, NTMF, and NICD both in vivo and in vitro	[37]
	trodusquemine	AβOs	selectively binds to oligomeric species and reduces the toxicity	[38]
	methylene blue	AβOs	inhibits the oligomer formation by selectively inducing the fibril formation	[39]
	sulforaphane	AβOs	decreases oligomer production, tau phosphorylation, oxidative stress, and inflammation, while enhancing cognition in PS1V97L Tg mice	[40]
	transthyretin (TTR)	AβOs	TTR tetramers inhibit Aβ aggregation in vitro through an interaction between the thyroxine binding pocket of the TTR tetramer and Aβ residues 18–21	[41]

peptidomimetic	RI-OR2	AβOs and AβFs	attaches itself to its corresponding region (KLVFF, residues 16−20) in native Aβ and disrupt Aβ self-association	[42]
	LPFFD	Aβ plaque and AβFs	the absence of a proton on the secondary substituted nitrogen in the peptide bond of the proline residue may impede the development of intramolecular hydrogen bonds inside fibrils	[43]
	D3	AβOs	D3 derivative peptides bind to Aβ in monomeric stage and stabilize these species within the diverse equilibria of Aβ assemblies, ultimately resulting in the eradication of AβOs	[44]
	APPI	Aβ plaque and AβFs	the binding of this 20-mer cyclic peptide to Aβ42 (in a 1:1 molar ratio) promotes the development of Aβ42 aggregates, thereby ameliorating Aβ42-mediated cellular toxicity	[45]

chaperon proteins	BRICHOS domain	Aβ plaque, AβOs, and AβFs	interferes with Aβ in the nucleation process and extends the lag phase	[46]
	HSP104	AβOs, protofibrils, and AβFs	the inhibition of Aβ fibrillization by Hsp104 is evident at Hsp104/Aβ, indicating a selective involvement of Hsp104 with aggregation intermediates (such as oligomers and protofibrils), during amyloid formation	[47]
	αB-crystallin	AβOs	αB-crystallin interacts with the monomer and oligomeric state of the proteins via capping the β-sheet elongation surfaces; it restricts the nucleation phase, which in turn does not allow the oligomer to form fibrils	[48]

### Emerging therapeutic approaches in clinical trials for targeting AβOs

Recent advancements have positioned immunotherapeutic approaches utilizing anti-Aβ antibodies as some of the most promising strategies for the treatment of AD. Notably, the first generation of anti-Aβ antibody therapies, including aducanumab, lecanemab, and donanemab, has demonstrated significant therapeutic potential in combating AD. Aducanumab and lecanemab have already received FDA approval, while donanemab is currently undergoing clinical evaluation [49]. In a noteworthy study by Sandberg et al., an oligomer-specific antibody known as ALZ 201 was reported to effectively mitigate the toxic effects associated with extracts from AD brains. This research confirmed that ALZ 201 selectively recognizes AβOs and, interestingly, demonstrated efficacy in protecting neurons exposed to AD brain extract. ALZ 201 is presently in preclinical development, highlighting its potential as a therapeutic candidate [50]. Lecanemab, another anti-Aβ antibody, has been found to exhibit a higher affinity for Aβ protofibrils characterized as “beaded” curvilinear fibrils and recognized as a specific form of AβOs than other known antibodies such as aducanumab or gantenerumab. Furthermore, clinical observations have indicated that treatment with lecanemab is associated with a reduction in cognitive decline, underscoring its promise as a viable therapeutic option in the fight against AD [49].

**Figure 3:**
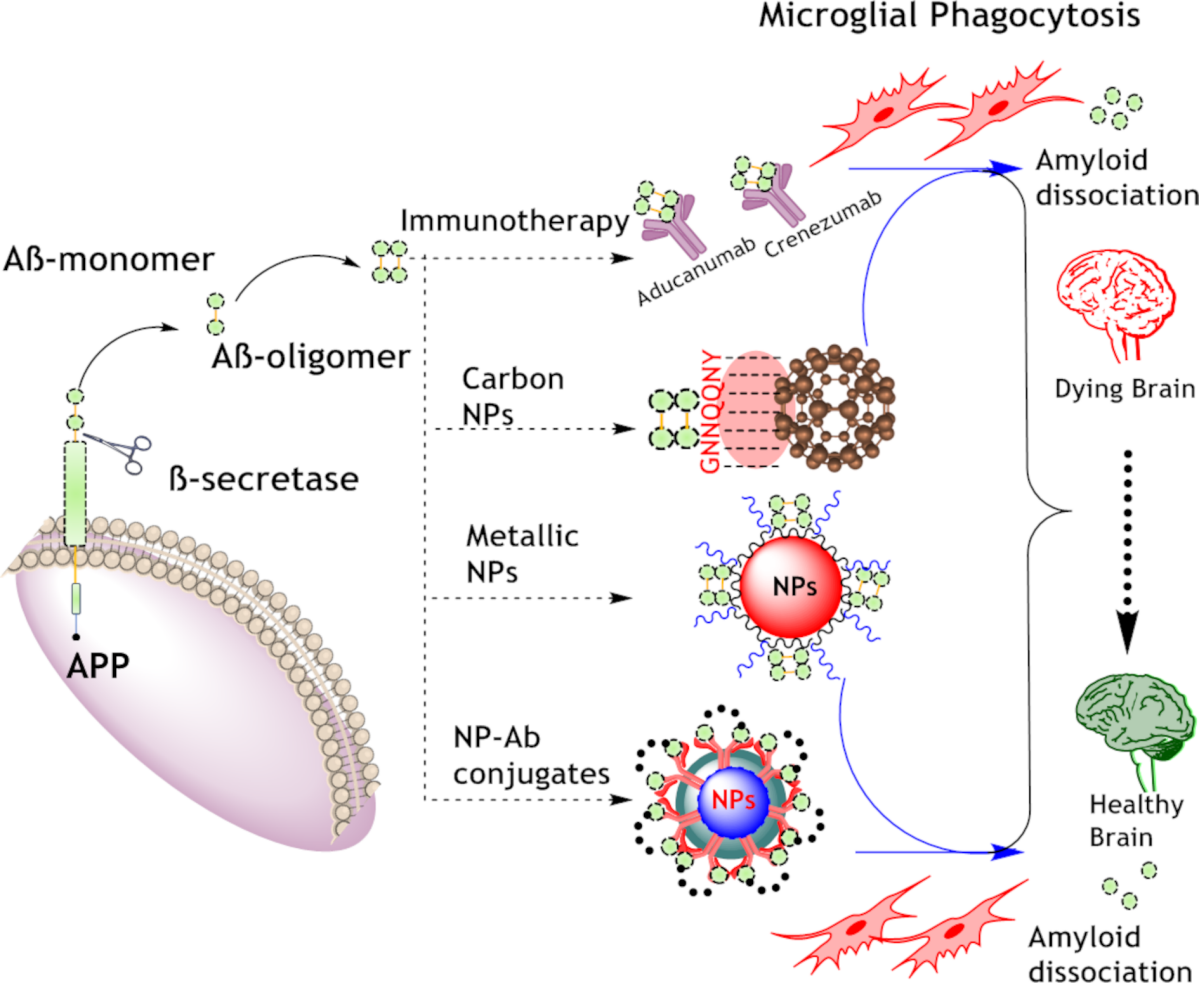
This figure summarizes the key strategies discussed in our review for targeting AβOs in AD. Aβ monomers, generated from APP through β-secretase activity, aggregate to form oligomers and subsequently amyloid plaques. The diagram highlights various therapeutic approaches, such as immunotherapy and antibody-conjugated NPs designed to enhance microglial phagocytosis for Aβ clearance, as well as NP-based techniques aimed at disrupting Aβ aggregates and preventing their toxic effects. (Figure 3 was created using ChemDraw Professional (Version 20.1.1.125) and Servier Medical Art (https://smart.servier.com)).

The landscape of AD treatment is evolving, with emerging therapeutic approaches in clinical trials targeting AβOs offering new hope for patients. Monoclonal antibodies and oligomer-specific antibodies are at the forefront of this research. As clinical trials progress, these therapies hold the potential to significantly improve cognitive outcomes and quality of life for individuals affected by AD, underscoring the importance of targeted interventions in combating this complex neurodegenerative disorder. Conventional approaches have shown promise in the detection and treatment of AβOs in AD. However, NP-based approaches present a complementary and potentially more versatile strategy for addressing AβOs in AD. In our review, we discuss a range of strategies aimed at targeting AβOs, and these approaches are illustrated in Figure 3. Figure 3 provides an overview of the different therapeutic strategies, highlighting both conventional and emerging methods for addressing the challenges posed by AβOs in AD pathology.

### Nanoparticle-based approaches for the diagnosis and dissociation/inhibition of AβOs

Although conventional approaches for diagnosing and targeting AβOs have laid the foundation for AD treatment, they often face limitations. For example, monoclonal antibodies, while capable of binding AβOs, may also interact with other forms of Aβ, including fibrils or monomers, leading to off-target effects and reduced efficacy. Recent advancements in nanotechnology offer a promising alternative with NPs specifically designed for AD diagnosis and AβO inhibition. These NPs possess unique properties, including variable size and shape and readily modifiable surfaces. These features allow for targeted and effective therapeutic strategies. In this section, we discuss NPs specifically designed for the diagnosis and inhibition of AβOs.

One example of this innovative approach is demonstrated by Viola et al., who designed mono-dispersed nitro-dopamine (nDOPA)- and polyethylene glycol (PEG)-stabilized magnetic nanostructures (MNSs) in a size range of 12–16 nm. The surface of these MNSs was further modified with oligomer-specific antibodies, creating a stable nanobioconjugate for both in vitro and in vivo applications. The MNSs could detect toxic AβOs present on nerve cell surfaces in vitro, demonstrating their specificity and effectiveness. Additionally, upon intranasal delivery in a mouse model, these MNSs rapidly targeted AβOs under in vivo conditions, providing strong MRI contrast, thus supporting their potential for non-invasive diagnostic imaging of early-stage AD [51]. In another study, a similar approach was utilized by Wang et al.; they developed a AβO-targeting gadolinium-based NIR/MR dual-modal theranostic nanoprobe. The developed nanoprobe was used as an efficient and sensitive MR/NIR contrasting agent for the detection of AβOs in different age groups of transgenic AD mice. The theranostic nanoprobe also showed strong inhibitory effect against Aβ fibrillation and improved associated neurotoxicity [52]. Shifting from imaging to electrochemical approaches, researchers have developed biosensors comprising immobilized thiolated PrPC peptides on a graphene oxide/gold nanoparticle hydrogel electrode. This nanobiosensor displayed high specificity and sensitivity for detecting soluble AβOs in artificial cerebrospinal fluid (CSF) or blood plasma. It was capable of effectively distinguishing AβOs from Aβ monomers and fibrils, indicating its utility for accurate and selective detection of AβOs [53]. Another group of researchers utilized a similar approach of electrochemical detection of AβOs via a nanobiosensor consisting of gold nanoparticles (AuNPs) embedded in a conductive polymeric matrix. For this, the surface of the AuNPs was further modified with PrPc, which acted as the biorecognition element for the specific detection of AβOs in ex vivo real samples, viz., CSF and blood tests [54]. Researchers have also employed the surface plasmon resonance (SPR) of citric acid-coated AuNPs, to specifically detect and quantify Aβ40 oligomers, as the SPR absorption band of AuNPs was found to be sensitive to the presence of AβOs [55]. While exploring the range of AβO detection methods, Liu et al. developed a fluorescence-based system using a FAM-labeled DNA aptamer fluorophore along with a nanoquencher attached to self-assembled polydopamine nanospheres. This nanosystem showed selective recognition of AβOs through a “fluorescence-signal on” mechanism, where the FAM-DNA aptamer interacted with AβOs, causing a hairpin-like conformational change that triggered a fluorescent signal. This approach combined sensitivity with specificity, providing a promising tool for the detection of AβOs [56]. While the aforementioned NP-based systems excel in identifying and imaging AβOs, these technologies also pave the way for developing therapeutic strategies aimed at inhibiting the formation of toxic AβOs. Liu et al. synthesized multifunctional superparamagnetic iron oxide nanoparticles (SPIONs) conjugated with a specific scFv antibody (W20) targeting AβOs and a class-A scavenger receptor activator (XD4). These W20/XD4-SPIONs demonstrated promising results in mitigating the cytotoxicity induced by AβOs and enhancing microglial phagocytosis of these toxic aggregates [57]. Previously, the same NPs were found to show promising early diagnostic potential for AD [58]. Brambilla et al. employed a combination of experimental and computational approaches to investigate the interaction between PEGylated NPs and Aβ monomers. Their findings revealed that surface interactions between NPs and Aβ monomers effectively inhibited the formation of AβOs [59]. Building upon their previous research, Parikh et al. developed a curcumin-loaded self-nanomicellizing solid dispersion system (Cur-SNSDS) to significantly enhance the in vivo bioavailability of curcumin. This novel NP system demonstrated superior safety and efficacy in mitigating Aβ42 oligomer-induced toxicity in SH-SY5Y695 APP human neuroblastoma cells compared to pure curcumin. Moreover, the Cur-SNSDS system effectively prevented cognitive decline in aged APPSwe/PS1deE9 mice, suggesting its potential as a therapeutic intervention for AD [60]. Ultimately, these diverse examples illustrate the transformative, versatile, and effective approach of NP-based strategies for the detection and inhibition of AβOs (Table 2). Their ability to target and interact with AβOs presents a highly promising avenue for future therapeutic development. As research continues in this area, NPs show promising potential to revolutionize how we diagnose and manage NDs such as Alzheimer’s.

**Table 2:** NP-based strategies for diagnosing and inhibiting AβO formation, as well as mitigating the associated toxicity.

Nanoparticles	Conjugation/modification	Mechanism of action	Reference

poly(dopamine) nanospheres	conjugated with carboxyfluorescein-labeled DNA aptamer	detection of AβOs with high sensitivity using carboxyfluorescein-labeled DNA aptamer–polydopamine nanospheres, capable of identifying concentrations as low as 20 nM	[56]
gold nanoparticles	casein-coated gold nanoparticles	chaperones together with gold nanoparticles effectively neutralized Aβ aggregates, reducing their toxic effects	[61]
iron oxide nanoparticles	conjugated with Aβ oligomer-specific scFv antibody W20 and class-A scavenger receptor activator XD4	mitigation of AβO-induced cell toxicity and stimulate microglial phagocytosis of Aβ	[57]
single-wall carbon nanotubes (SWCNTs)	hydroxylated SWCNTs	disruption of the beta-sheet conformation of Aβ16–22 oligomers, leading to the formation of less structured, disordered aggregates	[62]
PLGA NPs	—	interaction with AβOs during the elongation phase via π–π and hydrophobic interactions, destabilizing their harmful structure	[63]
gold nanoparticles	conjugated with oligomer specific antibody	detection of Aβ1–40 with exceptional sensitivity, capable of identifying concentrations as low as 1 fg/mL	[64]
PLGA NPs	conjugated with 83-14 mAb and encapsulated with rosmarinic acid (RA) and curcumin (CUR)	enhanced cellular uptake of RA and CUR when delivered using these nanocarriers, indicating that the antibody is crucial for improving nanoparticle delivery to the brain	[65]
exosomes (EXOs)	M2 microglia-derived exosomes	M2-EXOs were found to decrease Aβ plaque formation and Aβ oligomer expression in AD cell models, suggesting a protective role in AD pathogenesis through the enhancement of PINK1/Parkin-mediated mitophagy	[66]
AuNPs	conjugated with chiral ʟ- and ᴅ-glutathione	ᴅ-enantiomer showed a stronger binding affinity to Aβ42 and demonstrated improved reversal of behavioral deficits in mice modeling Alzheimer's disease	[67]
AuNPs	conjugated with glucosamine	the abundance of carbohydrate groups on the nanoparticle surface formed robust hydrogen bonds with protein oligomers, preventing their aggregation	[68]
PLGA NPs	conjugated with PEG and encapsulated with indirubin-3′-monoxime (I3M)	nanoparticles continuously released I3M, improving the ability to inhibit Aβ aggregation; additionally, PLGA-PEG nanoparticles enhanced the uptake of I3M by PC12 cells, demonstrating their potential to protect neurons from AβOs	[69]

In this review, we systematically categorize NPs used for the diagnosis and inhibition of AβOs based on their composition and functionalization. This bifurcation allows for a clearer understanding of the diverse mechanisms and applications of NPs in addressing AD. We have organized the NPs into four primary categories, namely, carbon based nanomaterials (CNMs), metal based NMs, biomimetic NMs and antibody-functionalized NMs.

#### Carbon-based nanomaterials for the detection and inhibition of AβO

Recent advances in nanomedicine have spotlighted CNMs because of their remarkable physicochemical properties, diverse structural forms, and potential applications in combating NDs. The unique characteristics of CNMs, including their hydrophobic surfaces and variable dimensions, enable them to interact effectively with biomolecules, making them valuable tools in biomedical research and therapeutic applications. CNMs can be categorized into three primary forms, namely, zero-dimensional fullerenes (e.g., C_60_), one-dimensional carbon nanotubes (CNTs), and two-dimensional graphene. Each of these NMs possesses distinct attributes that facilitate their engagement with proteins and peptides, particularly those associated with NDs like AD. Research has demonstrated the ability of fullerenes to prevent the aggregation of Aβ peptides. For instance, molecular dynamics simulations have shown that fullerenes inhibit the fibrillation of the hydrophobic KLVFFAE peptide by disrupting the formation of β-sheet oligomers. This property is particularly significant as β-sheet formation is a critical step in the aggregation pathway leading to neurotoxic amyloid fibrils [70]. Further investigations revealed that fullerene C_60_ interacts strongly with non-polar aliphatic groups in polar residues of the GNNQQNY peptide, effectively redirecting the formation of potentially toxic oligomers towards disordered coil structures. This mechanism not only hinders fibril formation but also shifts the balance toward less harmful aggregates [71]. Single-walled carbon nanotubes (SWCNTs) have emerged as another promising CNM for the detection and inhibition of AβOs. Studies indicate that hydroxylated SWCNTs significantly inhibit the β-sheet formation of Aβ peptides [17–23]. By facilitating the formation of disordered aggregates, these nanomaterials diminish the aggregation propensity of Aβ peptides, thereby mitigating their neurotoxic effects [62,72]. In addition to their inhibitory capabilities, SWCNTs can serve as effective sensors for AβOs. Their ability to interfere with β-sheet formation, a hallmark of Aβ aggregation, has been confirmed through comprehensive molecular dynamics simulations. These studies reveal that SWCNTs interact with the hydrophobic residues of Aβ peptides, particularly through π-stacking interactions with aromatic amino acids such as phenylalanine. This interaction destabilizes the prefibrillar β-sheet structures, preventing the formation of toxic oligomers and promoting the aggregation of less harmful conformations [73]. Moreover, the development of positively charged carbon quantum dots has shown promise in preventing the aggregation of amyloid proteins, specifically by inhibiting the formation of hetero-oligomers between islet amyloid polypeptide (IAPP) and Aβ42. Such findings highlight the versatility of CNMs in addressing different aspects of amyloid aggregation [74]. In summary, CNMs present a multifaceted approach to the detection and inhibition of AβOs. Their unique structural properties and interactions with amyloid peptides hold significant potential for the development of innovative therapeutic strategies aimed at combating NDs. As research in this field continues to advance, the integration of CNMs into clinical applications may offer new avenues for early detection and intervention in AD and related disorders.

#### Metal nanomaterials for detection and inhibition of AβOs

Metal NPs have emerged as pivotal tools in the detection and inhibition of Aβ1–42 oligomers. Their unique optical and electrical properties, particularly those of gold and silver NPs, enhance sensitivity and specificity in identifying the early stages of Aβ aggregation. By binding to AβOs, these NPs facilitate label-free detection methods such as SPR, colorimetric changes, and fluorescence amplification, enabling straightforward real-time monitoring of oligomer formation. This innovative approach not only deepens our understanding of amyloid pathology but also contributes to the development of diagnostic strategies for NDs. Zhou and colleagues introduced an advanced electrochemical aptasensor that utilizes metal-organic frameworks (MOFs) as signal probes for detecting AβOs. They engineered an electrode modified with gold nanoflowers to capture targets, employing aptamer-tagged gold nanoparticle/Cu-MOFs conjugates to produce sensitive signals. This resulted in a highly effective sandwich sensor capable of detecting AβOs in a linear range from 1 nM to 2 μM, demonstrating a correlation coefficient of 0.996 and a low detection limit of 0.45 nM [75]. Phan and team developed a robust and straightforward method for creating multichamber paper devices using wax printing techniques, which they applied to detect AβOs. This approach leverages copper-enhanced gold nanoprobe colorimetric immunoblotting, achieving detection limits as low as 23.7 pg/mL, visible through a smartphone camera, and up to 320 pg/mL with the naked eye [76]. Zhao and collaborators utilized AuNPs embedded in various matrices to construct three-dimensional layers for detecting AβOs. Among their innovations, PrPC/AuNPs embedded in a Ppy-3-COOH matrix (AuNPs-E-Ppy-3-COOH) exhibited superior sensitivity, with a detection range spanning from 10^−9^ to 10^3^ nM [77]. An electrochemical, label-free aptassay developed by Gallo-Orive et al. incorporates graphene oxide–gold nanoparticles/nickel/platinum nanoparticles for the rapid and accurate detection of AβOs in complex clinical samples, such as brain tissue and CSF from Alzheimer’s patients. This method showcased exceptional sensitivity with a limit of detection of 0.10 pg/mL, demonstrating reproducibility and rapidity [78].

**Figure 4:**
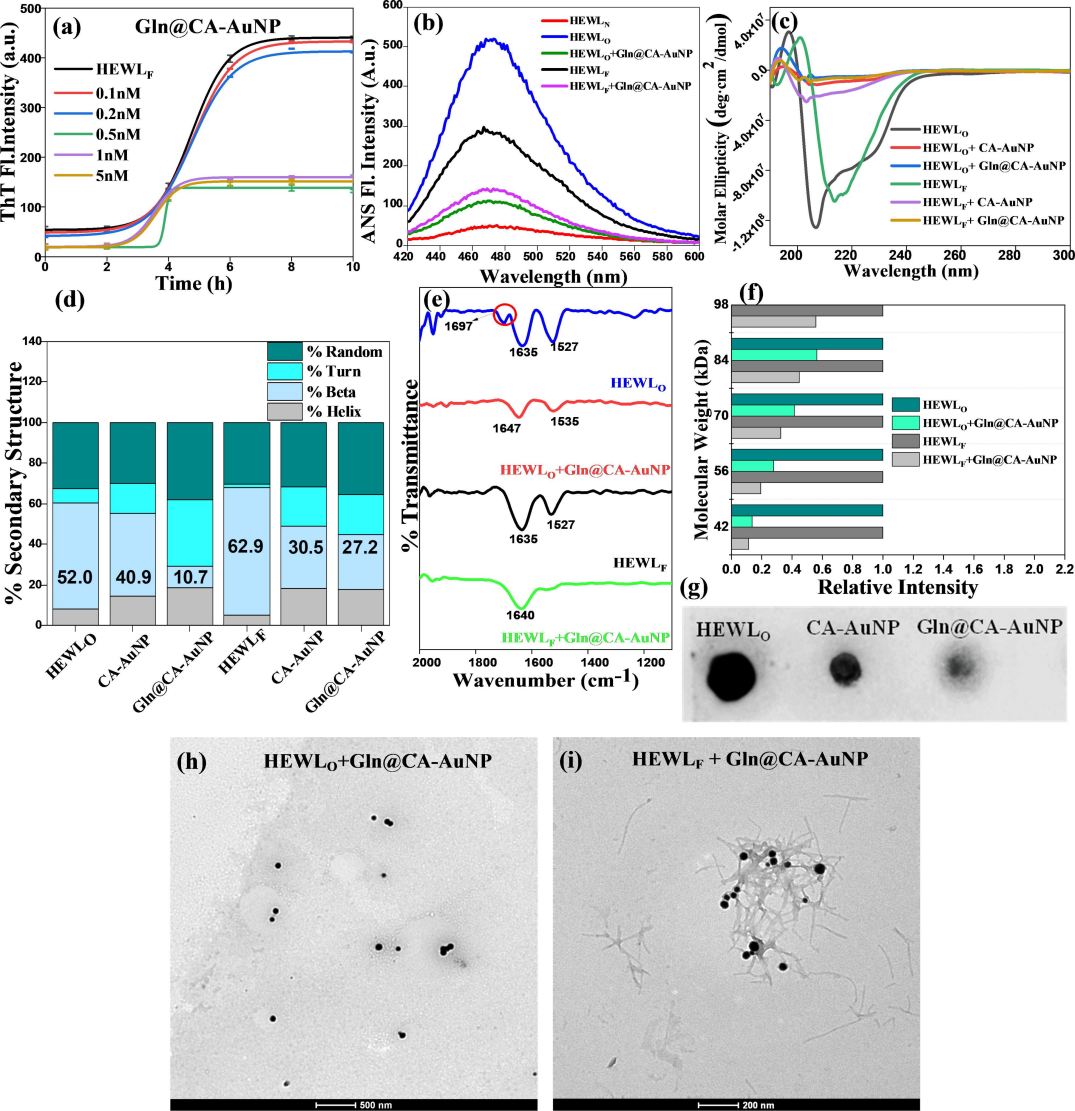
This figure compares the inhibitory effects of Gln@CA-AuNP on HEWL_O_ and HEWL_F_ aggregation demonstrating effective inhibition and potential disruption of oligomer formation. (a) Thioflavin T (ThT) assay, (b) 8-anilinonaphthalene-1-sulfonic acid (ANS) fluorescence, (c) circular dichroism spectra, (d) β-sheet content, (e) FTIR, (f) MALDI-TOF, and (g) dot blot with A11 antibody. TEM images (h) HEWL_O_ and (i) HEWL_F_ show reduced aggregation after Gln@CA-AuNP treatment. Gln@CA-AuNP inhibits HEWL_O_ aggregation more effectively than that of HEWL_F_. (Figure 4 was reprinted from [68], Nano Today, vol. 56, by S. Randhawa; A. I. Dar; T. C. Saini; M. Bathla; A. Acharya, “Glucosamine conjugated gold nanoparticles modulate protein aggregation induced autophagic neuronal cell death via regulation of intracellular Parkin homeostasis“, article no. 102243, Copyright (2024), with permission from Elsevier. This content is not subject to CC BY 4.0).

Metallic NPs have gained considerable attention as potential therapeutic agents regarding AβO formation. Their unique surface characteristics enable specific interactions with amyloid fibrils, effectively inhibiting oligomerization and reducing neuronal cell death. Moreover, these NPs can be functionalized with targeted ligands, enhancing their selectivity and efficacy in therapeutic applications aimed at Aβ-induced neurotoxicity. Recent research has focused on cyclometallated palladium complexes (Pd-1, Pd-2, and Pd-3), which incorporate anthracene and pyrene within a tridentate ligand framework. These complexes specifically target the oligomerization of soluble Aβ1–42 peptides. Among them, Pd-3 has shown significant promise, exhibiting the greatest reduction in Aβ1–42 peptide-induced cytotoxicity in Neuro-2a cell lines. Structural studies indicate that these palladium complexes interact with both the fibrillar (PDB: 2BEG) and monomeric (PDB: 1IYT) forms of the Aβ1–42 peptide. This interaction occurs through a variety of binding modalities, including hydrophobic and hydrogen bonding, leading to substantial inhibition of peptide aggregation [79]. In another innovative approach, Javed et al. evaluated the inhibitory potential of casein-coated AuNPs against oligomers through molecular dynamics simulations. Their findings demonstrated that these NPs effectively bind to oligomeric species, preventing the formation of fibrillar structures [61]. The influence of varying diameters and lengths of cetyltrimethylammonium bromide-stabilized gold nanorods (AuNRs) on Aβ oligomerization and fibrillation has also been thoroughly investigated. Fluorescence studies revealed that the presence of the AuNRs significantly inhibits the development of larger oligomers and fibrils, with inhibition efficacy diminishing as the diameter of the NPs decreases [80]. In a different approach, Randhawa et al. designed glucosamine-conjugated gold nanoparticles (Gln@CA-AuNP), which demonstrated strong inhibition of hen egg-white lysozyme oligomers (HEWL_O_) in comparison to fibrils (HEWL_F_) (Figure 4). The high density of carbohydrate moieties on the NP surface facilitated strong hydrogen bonding with protein oligomers, preventing their aggregation. Additionally, Gln@CA-AuNP was found to enhance the production of sulfated glycosaminoglycans, bolster extracellular matrix generation, and confer neuroprotection against oligomeric protein aggregates [68]. These findings collectively underscore the potential of metal-based NPs in not only inhibiting Aβ oligomerization but also in paving the way for effective therapeutic strategies against NDs.

#### Biomimetic nanomaterials based on cell primitives for targeting AβOs

**Figure 5:**
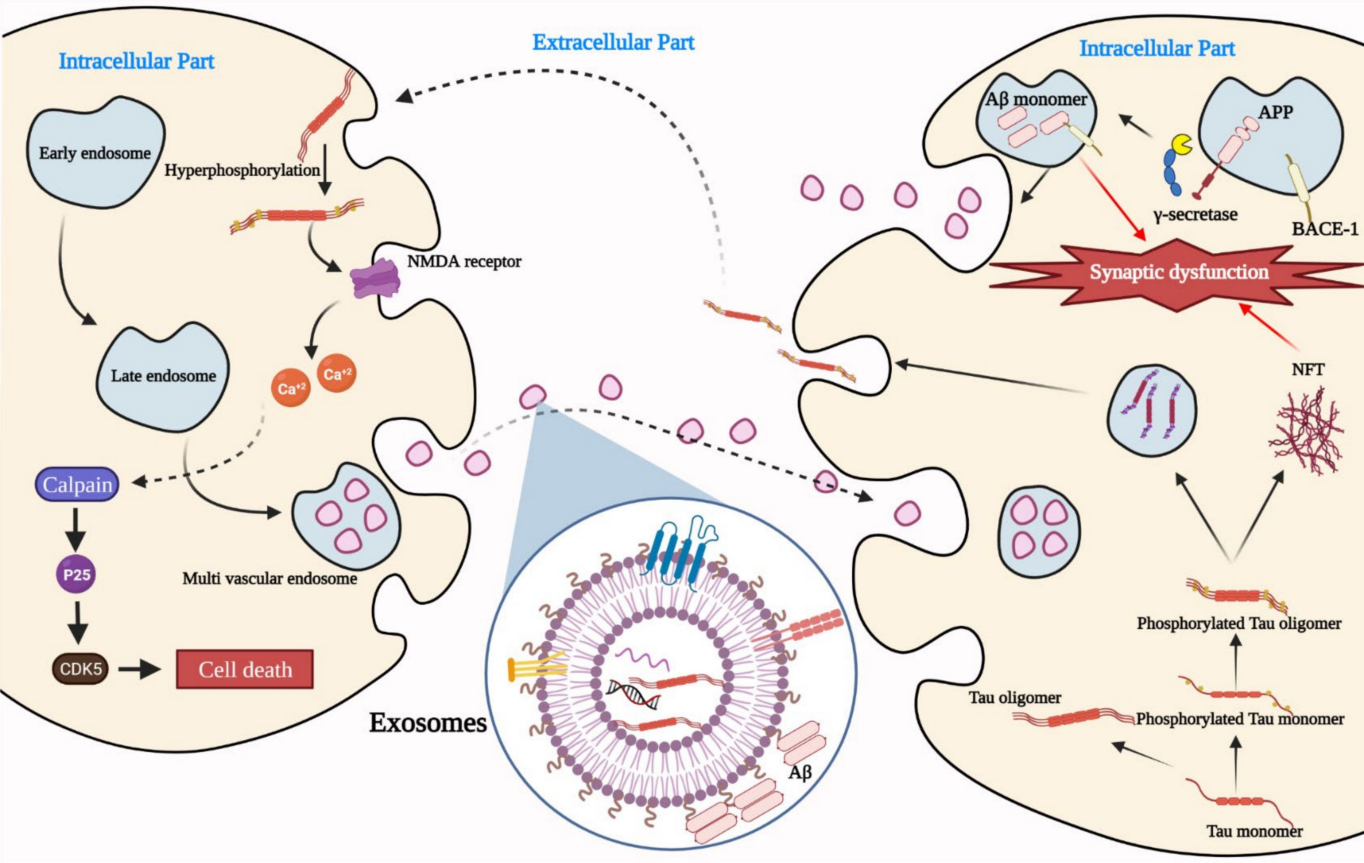
Illustration of the role of exosomes in transporting AβOs and tau protein, which are implicated in the accumulation associated with AD. This highlights the potential of exploiting exosomes for diagnostic purposes in detecting these pathological markers. (Figure 5 was reproduced from [83] (© 2021 Hagar M. Soliman et al., published by MDPI, distributed under the terms of the Creative Commons Attribution 4.0 International License, https://creativecommons.org/licenses/by/4.0/)).

The treatment of AD using conventional pharmacological agents has encountered significant challenges, prompting researchers to explore multifunctional nanobiomaterials derived from cell primitives as a promising therapeutic strategy. These biomimetic nanomaterials, comprising components such as cells, extracellular vesicles (EVs), and cell membranes offer several advantages. Their nanoparticulate size facilitates long-term circulation, reduces immune response, enables targeted delivery to lesion sites, and retains distinct biological functions. Emerging studies have highlighted the role of exosomes derived from AD brain samples, which contain elevated levels of AβOs. These exosomes are implicated in the propagation of AβOs between neurons, suggesting their potential utility as diagnostic biomarkers for early AD detection [81–82]. Researchers are exploring the potential of exosomes as diagnostic tools, leveraging their ability to carry these pathological proteins. Exosomes hold significant diagnostic potential because of their nanoscale size and the distinct profile of biomolecules they carry, which reflect the characteristics of their parent cells. These biomolecules, including proteins, nucleic acids, and lipids, serve as a “fingerprint” of the originating cells. When cellular conditions change, such as during disease progression, alterations in the composition of exosomes can provide valuable insights into the underlying pathology, making them a promising tool for diagnostic applications. Figure 5 demonstrates how exosomes can transport AβOs and tau protein, both of which are key biomarkers associated with AD. The accumulation of these proteins is central to the pathophysiology of AD, and their presence in exosomes can aid in the diagnosis of the disease. By analyzing the presence and levels of AβOs and tau protein in exosomes derived from biological fluids, such as blood or CSF, it may be possible to develop non-invasive diagnostic methods for the early detection of AD [83]. However, recent research has begun to investigate the therapeutic potential of exosomes themselves in mitigating AβO-induced toxicity. For instance, Bodart-Santos et al. isolated and characterized EVs from human Wharton’s jelly mesenchymal stem cells (hMSC-EVs) and assessed their neuroprotective effects in primary hippocampal cultures exposed to AβOs. Their results indicated that hMSC-EVs could protect neurons from AβO-induced damage, largely attributed to the transfer of enzymatically active catalase contained within the EVs. This approach holds promise for developing cell-free therapeutic strategies for AD [84]. Additionally, Deng et al. explored exosomes isolated from ultrasound-stimulated human astrocytes (US-HA-Exo). Characterization via nanoparticle tracking and proteomic analysis revealed that ultrasound treatment increased exosome release approximately five-fold. These exosomes exhibited rapid internalization in SH-SY5Y cells and co-localized with FITC-conjugated AβOs. Furthermore, CCk-8 assays demonstrated that US-HA-Exos could alleviate AβO toxicity in vitro [85]. Li et al. investigated M2 microglia-derived exosomes (M2-EXOs) and their impact on AD progression. They found that treatment with M2-EXOs in AD cell models, such as HT-22- and MAP2-positive neuronal cells, significantly reduced Aβ plaque deposition and expression of AβOs. Their findings suggest that M2-EXOs confer protective effects in AD pathogenesis through the modulation of PINK1/Parkin-mediated mitophagy [66]. Moreover, Yuyama et al. reported that exosomes secreted by neuronal cells inhibit Aβ oligomerization by enhancing microglia-mediated Aβ clearance in vitro [86]. In a different approach, Senapati et al. developed a multifunctional liposome-based platform incorporating a novel cyclic peptide (CP-2) that selectively targets toxic AβOs. Their studies indicated that CP-2-liposomes effectively disrupt Aβ aggregation, mitigate Aβ-mediated toxicity, and improve cognitive and behavioral outcomes in both in vitro and in vivo models. Notably, these liposomes can cross the BBB, suggesting their potential for precise diagnosis and targeted treatment of AD [87]. In conclusion, biomimetic nanomaterials derived from cell primitives show great promise in addressing the complexities of AβO-related toxicity in AD. Continued research into these innovative approaches could pave the way for effective therapeutic interventions and early diagnostic tools in the fight against AD.

#### Antibody-functionalized nanomaterials for detection and inhibition of AβOs

Unmodified NMs often demonstrate limited selectivity and functionality for AβO detection and inhibition, potentially leading to false positives or inadequate therapeutic outcomes. To enhance their efficacy, NMs can be conjugated with specific ligands or molecules. Antibodies, highly specific proteins that bind to unique epitopes on AβOs, provide precise targeting, minimizing off-target effects and maximizing therapeutic efficacy. Their high affinity and specificity allow for efficient capture even at low concentrations, crucial in the complex biological environment of the brain. Recent advancements in immunoassay technology have led to the development of ultrasensitive methods for detecting Aβ-derived diffusible ligands (ADDLs) and AβOs. A novel immunoassay, capable of detecting ADDLs at femtomole levels, has been developed [88]. This assay is instrumental in understanding the formation of oligomers in vivo as it allows for the detection and quantification of these crucial intermediates in the aggregation process. Zhou et al. introduced an antibody–aptamer sandwich assay utilizing gold nanoparticles for sensitive detection of AβO, achieving a low detection limit of 100 pM, which is crucial for early AD diagnosis [89]. A novel SPR-based immunosensor, enhanced with gold NPs and antibodies, was developed for ultrasensitive detection of Aβ1–40. The sensor demonstrated a linear response over nine orders of magnitude, with a detection limit of 1 fg/mL. This approach holds promise for early diagnosis and treatment of amyloid-related diseases [64].

Moreover, antibodies can facilitate the uptake of NPs by cells, a crucial step for delivering therapeutic payloads to intracellular targets like amyloid oligomers within neurons. Antibody-coated PEGylated NPs have been shown to break down Aβ42 and may successfully minimize neurotoxicity caused by Aβ fibrils in the brain. Kuo et al. developed novel NPs composed of poly(acrylamide)-cardiolipin (CL)-poly(lactic-*co*-glycolic) acid (PLGA) and grafted with surface 83-14 monoclonal antibody (MAb). These NPs were designed to deliver rosmarinic acid (RA) and curcumin (CUR) across the BBB and improve the viability of SK-N-MC cells damaged by amyloid deposits. The researchers found that increasing the concentration of 83-14 MAb on the NPs led to a higher permeability of RA and CUR across the BBB. This suggests that the antibody plays a crucial role in improving the uptake of NPs by cells and drug delivery to the brain [65]. Additionally, antibody conjugation can help to reduce the immunogenicity of NPs, enhancing their safety and tolerability. By combining the unique properties of NPs with the specificity and targeting capabilities of antibodies, these conjugates offer a powerful approach to enhance the specificity, sensitivity, and functionality of diagnostic and therapeutic tools for amyloid oligomers. Many immunotherapies targeting amyloid oligomers have led to side effects such as hydrocephalus, inflammation, and amyloid-related imaging abnormalities [90]. These side effects can be attributed to the activation of the complement system by the interaction of amyloid oligomers and antibodies, leading to the release of proinflammatory fragments. Additionally, complement fragments can further activate microglia, exacerbating neuroinflammation [91]. The effector fragment of the antibody plays a crucial role in these side effects. The ScFv antibody W20, which is specific for amyloid oligomers and lacks the effector fragment, can potentially eliminate these adverse effects. Multifunctional SPIONs conjugated with W20 and XD4, a class A scavenger receptor activator not only have diagnostic value but also retain the anti-amyloid properties of W20 and XD4, inhibiting amyloid aggregation, reducing cytotoxicity, and enhancing microglial phagocytosis. When administered to APP/PS1 mice, these NPs significantly improved cognitive function and reduced neuropathology associated with AD [58]. Carradori et al. investigated the therapeutic efficacy of antibody-functionalized polymer NPs for AD. In an AD-like transgenic mouse model, these NPs demonstrated significant reductions in brain AβO levels and concomitant improvements in memory function. The NPs were designed to interact with Aβ1–42 peptides in the bloodstream, promoting their elimination via a “sink effect”. Treatment with anti-Aβ1–42-functionalized NPs resulted in a complete reversal of memory deficits, a substantial decrease in soluble Aβ and oligomer levels within the brain and a marked increase in Aβ levels in the plasma, indicating enhanced clearance from the body. These findings highlight the potential of antibody-functionalized NPs as a promising therapeutic strategy for AD [92]. While the use of NPs with antibodies shows great potential, challenges remain in ensuring specificity and minimizing potential immunogenic responses in therapeutic applications.

### Factors affecting the therapeutic potential of NP-based AD therapeutic agents

The anti-oligomeric efficacy of NPs depends on their size, shape, and surface properties, as these factors collectively influence how they interact with amyloidogenic proteins. Smaller NPs can infiltrate and break early-stage oligomers, whereas differences in shape influence binding efficiency and selectivity. Surface functionalization, such as hydrophobic modification, enhances the targeting of β-sheet-rich oligomers and helps to prevent further aggregation. The unique features of NPs make them promising candidates for inhibiting amyloid production in NDs. Shape and structure of nanoparticles play a crucial role in how they interact with amyloid oligomers, affecting their binding ability and preventing aggregation. Different nanoparticle geometries facilitate specific surface interactions that help to block oligomer formation and support structural stability. For example, Xiong et al. showed that peptide-functionalized AuNPs could effectively inhibit amyloid fibrillation. By attaching two peptide inhibitors (VVIA and LPFFD) to the surface of AuNPs using Au–S bonds, they created hybrid AuNPs that prevented oligomerization and reduced β-sheet formation, promoting random coil structures instead. The combined effect of these hybrid AuNPs was stronger than using the individual peptides alone, demonstrating the enhanced potential of nanoparticle-based treatments [93]. Building on these findings, Kim et al. showed that both the size and shape of AuNPs significantly affect Aβ aggregation. Their study revealed that smaller 20 nm AuNPs facilitated the formation of protofibrils and remained well-dispersed within Aβ aggregates, while larger 50 and 80 nm AuNPs promoted the formation of larger, plaque-like structures. This was due to increased Aβ accumulation on the surface of the larger nanoparticles, which accelerated aggregation through localized concentration effects. Additionally, they examined the impact of nanoparticle shape and found that gold nanocubes led to larger Aβ aggregates compared to AuNRs, likely because of their greater surface area and more uniform structure. In contrast, spherical AuNPs disrupted β-sheet stacking, resulting in less stable aggregates. These findings emphasize that nanoparticle size and shape are key factors in controlling amyloid aggregation, providing valuable insights for designing nanoparticle-based therapies for AD [94]. Beyond size and shape, surface functionalization plays a crucial role in governing nanoparticle interactions with amyloidogenic proteins, influencing aggregation pathways and inhibition efficiency. Moore and colleagues investigated how 18 nm AuNPs with different surface coatings, viz., citrate, polyallylamine (PAH), and polyacrylic acid (PAA), affected the aggregation of Aβ1–40 monomers. Their study found that PAA-coated nanoparticles completely inhibited aggregation, even at substoichiometric concentrations, while citrate- and PAH-coated nanoparticles reduced aggregation by 19% and 59%, respectively [95].

In contrast, John et al. used Thioflavin T fluorescence assays to explore the size-dependent effects of AuNPs on the fibrillogenesis of four peptides, namely, Aβ40, NNFGAIL, GNNQQNY, and VQIYVK. Their results showed that 5 nm AuNPs effectively inhibited or delayed fibril formation in Aβ40, GNNQQNY, and VQIYVK, but had no significant effect on NNFGAIL. In contrast, larger 20 nm AuNPs either accelerated fibril formation or had little impact on peptide aggregation [96]. In conclusion, these studies underscore the critical role of nanoparticle size, shape, and surface chemistry in modulating amyloid aggregation and inhibition. Smaller nanoparticles, particularly those around 5 nm in size, demonstrated superior inhibition by effectively interfering with early-stage oligomer formation. Additionally, surface functionalization significantly impacted aggregation dynamics, with different coatings (such as PAA, PAH, and citrate) showing different inhibitory effects. Structural variations, including the shape of nanoparticles, further influenced their ability to modulate amyloid fibrillogenesis. These findings highlight the importance of carefully designing nanoparticle-based strategies, considering size, shape, and surface modifications, to effectively inhibit oligomerization and provide potential therapeutic approaches for Alzheimer’s disease and other neurodegenerative conditions.

### Distribution and clearance of NPs

Because of their small size and high surface area-to-volume ratio, NPs have the ability to cross biological barriers such as skin and BBB, which makes them valuable candidates for various biomedical applications such as diagnosis, therapeutics, and drug delivery. The efficacy of these NPs is closely linked to their pharmacokinetic properties. The biodistribution, safety profile, and clearance mechanism of NPs plays a critical role in the clinical application of these NPs. Therefore, complete understanding of distribution and clearance of NPs in vivo is important. Following intravenous injection, NPs circulate through the bloodstream and are distributed to various organs and tissues (e.g., lungs, liver, kidney, and brain) based on their properties. Distribution is followed by their clearance, which mainly occurs through two pathways. The first pathway is the reticuloendothelial system (RES), and the second pathway is the renal and hepatic system [97–98]. The RES phagocytizes NPs and helps in their clearance. The renal and hepatic system eliminates NPs through kidneys and liver and excretion in urine and fecal matter, respectively. In the brain, the biodistribution of NP mainly occurs through the CSF. Direct infusion into the CSF results in rapid and widespread NP distribution, particularly in brainstem, cerebellum, and amygdala. The clearance of NPs from the brain is predominantly mediated by the paravascular glymphatic pathway, with studies suggesting that up to 80% of NPs are cleared through this route. Biodistribution and clearance of NPs have been shown to depend on their size and surface properties. Smaller graphene oxide (GO) sheets (20–70 nm) have been primarily distributed in kidney, spleen, and liver, while larger GO sheets (>200 nm) accumulate in the lungs [99]. For brain targeting, NPs between 100 and 300 nm have been found optimal for BBB transport, while those smaller than 15 nm show faster clearance. Surface characteristics also influence clearance; uncoated NPs have been rapidly cleared via the RES, whereas PEG-coated NPs exhibited extended circulation time and reduced RES clearance [99]. These findings highlight the importance of engineering nanoparticle size and surface properties to optimize therapeutic efficacy and clearance mechanisms. Achieving an ideal balance between prolonged circulation time, targeted delivery, and efficient clearance is crucial for maximizing the clinical potential of NPs.

### Toxicity concerns of NPs and their implications on health

The prolonged retention and low clearance of NPs in the body may result in extended exposure, leading to their accumulation in various tissues and organs, such as liver, kidneys, brain, and lungs. This accumulation can cause both local and systemic toxicity. Additionally, NPs have the potential to trigger immune responses, including inflammation and allergic reactions, by activating macrophages and other immune cells [100]. The size and surface characteristics of NPs are critical in determining their interactions with cells, which may lead to oxidative stress, inflammation, and disruption of normal cellular processes. These effects can result in DNA damage, protein denaturation, and lipid peroxidation, potentially contributing to the development of chronic diseases [101]. The cumulative cellular damage and inflammatory responses associated with NP exposure may give rise to long-term health complications. For instance, zinc oxide NPs have been shown to induce oxidative stress and DNA damage in mice models, along with alterations in various liver enzymes [102]. Similarly, SiO_2_ NPs generated by Lin et al. caused toxicity in human bronchoalveolar cells through an increase in ROS levels. In addition to ROS, SiO_2_ NPs also induce inflammation by upregulating inflammatory markers such as IL-1, IL-6, IL-8, and TNF-α, and causing mitochondrial damage [103]. NPs have the ability to cross the BBB and thus impact brain functions, which also makes them useful for treating neurodegenerative diseases. Upon crossing the BBB, NPs tend to accumulate in specific regions of the brain, where they can interact with neural cells, including neurons, astrocytes, and microglia. For example, iron oxide NPs can influence synaptic transmission, nerve conduction, neural inflammation, apoptosis, antioxidant responses, and immune cell infiltration [104]. Additionally, certain NPs, such as silver and copper oxide NPs, can disrupt the BBB, facilitating the entry of harmful substances into the brain, which may contribute to neurodegeneration [105]. The toxicological effects of NPs are further complicated by the fact that their long-term biological impacts are not fully understood. Given the complexity of their interactions with biological systems, NP behavior can vary based on factors such as size, shape, surface charge, and coating, making it challenging to predict potential health risks accurately. To mitigate these risks, surface modifications, such as coating NPs with biocompatible materials like polymers or lipids, can enhance their stability and prevent adverse cellular interactions. For instance, PEG modifications can help NPs evade the mononuclear phagocyte system, thus reducing toxicity [106]. Mesoporous SiO_2_ nanoparticles (MSNs) with thiol surface modifications have been shown to reduce oxidative stress and cellular damage significantly compared to unmodified MSNs, thereby improving biocompatibility [107]. Furthermore, optimizing size and shape of NPs can further minimize harmful effects, and incorporating antioxidant and anti-inflammatory coatings can mitigate oxidative stress. For example, smaller NPs (<50 nm) with neutral or slightly negative surface charges diffuse more efficiently through brain white matter with reduced toxicity [108]. Targeted drug delivery systems can direct NPs to specific brain regions, reducing the risk of unintended exposure, while biodegradable NPs, such as those made from PLGA, degrade into non-toxic byproducts and reduce long-term accumulation in neural tissues [109]. Additionally, functionalizing NPs with specific ligands or receptor-targeting molecules, like polysorbate 80, can selectively target inflamed or diseased areas of the brain, minimizing off-target effects and systemic toxicity [110]. Additionally, promoting the clearance of NPs through the brain’s waste removal systems, such as the glymphatic system, and developing NPs that mimic natural biological structures, could further improve safety and efficacy. These innovations provide a pathway for harnessing the potential of NPs in brain-targeted therapies, reducing toxicity, and maximizing therapeutic benefits. Therefore, while NPs hold significant promise for medical applications, their biological toxicity presents a considerable concern that necessitates rigorous regulation and continued scientific investigation.

### Future perspectives

**Figure 6:**
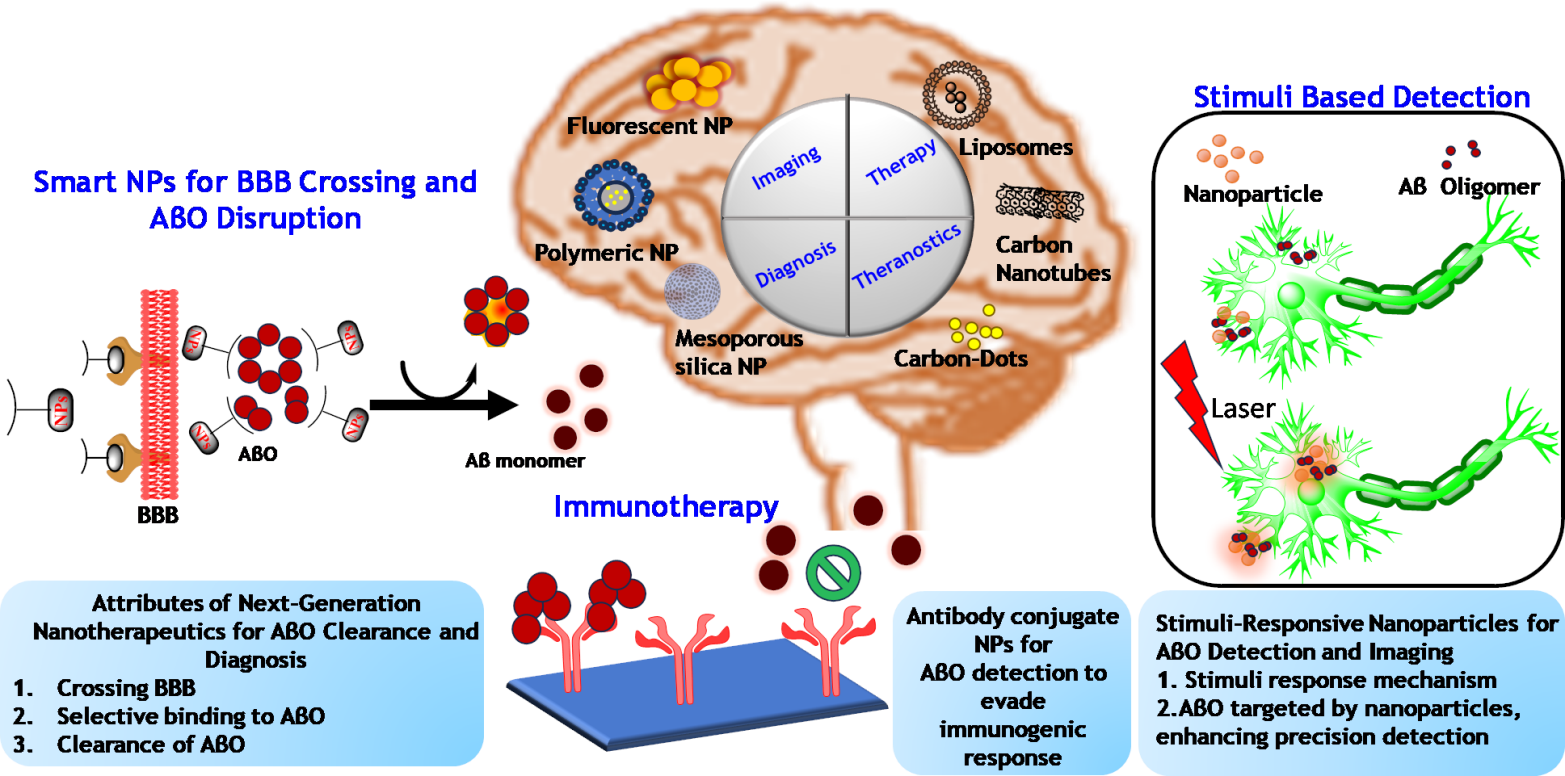
Nanotechnological strategies for diagnosing, targeting, and imaging AβOs to halt the progression of Alzheimer's disease. (Figure 6 was created using Microsoft PowerPoint and ChemDraw Professional (Version 20.1.1.125)).

Current therapeutic approaches for AD have focused on developing clinical candidates that specifically target AβOs. Targeting AβOs has several advantages, primarily the early appearance of AβOs in AD patients, making them a critical focus for intervention. The foremost challenge is to create drug candidates with superior specificity and selectivity for AβOs alone. Additionally, it is essential to distinguish AβOs from other chemically similar and more abundant forms of the peptide, which complicates treatment strategies. Traditional methods for targeting AβOs, such as small-molecule inhibitors and passive immunization, have shown promise but often fall short in efficacy and specificity. Fortunately, NPs offer a promising solution for both the detection and disaggregation of AβOs and fibrils. Their unique characteristics, including small size, ease of surface modification, and multivalency effects, provide a potential pathway for therapeutic intervention in AD. These strategies can be categorized based on their composition and functionalization, enabling a diverse range of applications tailored to specific therapeutic needs. CNMs and metal-based NPs have shown remarkable potential in detecting and inhibiting AβO aggregation, demonstrating their versatility in both research and clinical settings (Figure 6).

Moreover, biomimetic nanomaterials derived from cell primitives present an exciting frontier, leveraging natural biological processes for more effective targeting and treatment of AβOs. Nevertheless, challenges remain, including the need to evaluate the biocompatibility and toxicity of NPs and to develop scalable production processes.

## Conclusion

The oligomer hypothesis has emerged as a leading explanation for the neurotoxicity observed in AD, highlighting AβOs as the major toxic species. AβOs are small, metastable aggregates with unique structural properties, including a higher β-sheet content, which complicates their targeting with conventional therapies. Their heterogeneity and transient nature present significant challenges in developing effective treatments. However, nanomaterial-based strategies offer a promising approach for detecting and inhibiting AβOs. Nanomaterials, because of their unique size and surface properties, provide several advantages, including the ability to cross the BBB, increased surface area for functionalization, and potential for high specificity in targeting AβOs. However, despite these promising attributes, significant challenges remain such as limited efficacy and specificity in targeting the diverse and dynamic forms of AβOs, as well as the potential for off-target effects. These limitations can be overcome by optimizing the size, shape, and surface functionalization of NPs. Such efforts hold the potential to significantly improve the diagnosis, treatment, and management of AD, offering a transformative approach for addressing NDs.

## Data Availability

Data sharing is not applicable as no new data was generated or analyzed in this study.
